# Supplementary material for: A Critical Review on the Dosing and Safety of Antifungals Used in Exotic Avian and Reptile Species
Source: J Fungi (Basel). 2023 Jul 31;9(8):810. doi: 10.3390/jof9080810 (PMC10455840; doi:10.3390/jof9080810)
Supplement: Supplementary file 1 [file jof-09-00810-s001.zip › jof-2453041-supplementary.pdf]

Review

A Critical Review on the Dosing And Safety of Antifungals Used in Exotic Avian And Reptile Species

Naresh Visvanathan [1], Jolise Lim Yi An [1], Hui Ting Chng [1,*] and Shangzhe Xie [2]

Supplementary Materials:

Table S1. Efficacy and safety parameters of commonly reported antifungals for avian species.

Species	ROA	Dosing regimen	Formulation	Causative pathogen	SOI	MIC (μg/mL)	Efficacy	Treatment outcome	Adverse effects	Ref.
**AZOLES**										
Clotrimazole										
African grey parrot (n=1)	NEB	10 mg/mL 30-45 min SID on 3 day on, 2 day off schedule x 2 months	Dissolved in PEG 300	*Aspergillus spp.*	Respiratory tract	NR	✓	Combination therapy with itraconazole 10mg/kg q24h x 1 month but it was upon clotrimazole nebulisation when rapid clinical improvements were observed. Attitude, activity level, and respiratory functions visibly improved and WBC count went back to a normal range.	No adverse effects were observed.	[1]
Citron-crested cockatoo (n=1)	NEB	10 mg/mL 30-45 min SID on 3 day on, 2 day off schedule once weekly x 8 weeks	Dissolved in PEG 300	*Aspergillus spp.*	Respiratory tract	NR	✓	Combination therapy with flucytosine 120mg/kg QID x 2 weeks but it was upon clotrimazole nebulisation when rapid clinical improvements were observed. Attitude, activity level, and	Only mild discomfort and regurgitation during first treatment were observed along with rapid blinking	[1]

								respiratory functions visibly improved and WBC count went back to a normal range.	frequently during nebulisation.	
Gyrfalcon (n=1)	NEB	10 mg/mL 30-45 min SID on 3 day on, 2 day off schedule x 4 months	Dissolved in PEG 300	*Aspergillus spp.*	Respiratory tract	NR	✓	Combination therapy with itraconazole 10mg/kg q24h x 1 month but it was upon clotrimazole nebulisation when rapid clinical improvements were observed. Attitude, activity level, and respiratory functions visibly improved and WBC count went back to a normal range.	No adverse effects were observed.	[1]
Micronesian kingfisher (n=1)	NEB	40mg x 1h a day	1% solution diluted in 4mL water	*Aspergillus spp.*	NR	NR	✓	Appetite, body weight and attitude returned to normal. Normal white blood count with resolved monocytosis. Lack of significant lesions.	No adverse effects were observed.	[2]
Red-tailed hawk (n=1)	NEB	10 mg/mL 30-45 min SID on 3 day on, 2 day off schedule x 3 months	Dissolved in PEG 300	*Aspergillus spp.*	Respiratory tract	NR	✓	Combination therapy with itraconazole 10mg/kg q24h x 1 month but it was upon clotrimazole nebulisation when rapid clinical improvements were observed. Attitude, activity level, and respiratory functions visibly improved and WBC count went back to a normal range.	No adverse effects were observed.	[1]
Yellow-naped	NEB	10 mg/mL 30-45 min SID on 3 day	Dissolved in PEG 300	*Aspergillus spp.*	Respiratory tract	NR	✓	Combination therapy with itraconazole 10mg/kg q24h x 9 days	Mild discomfort and regurgitation during	[1]

Amazon (n=1)		on, 2 day off schedule x 4 months						and amphotericin B nebulisation (1mg/mL of saline BID) x 21 days but it was upon clotrimazole nebulisation when rapid clinical improvements were observed. Attitude, activity level, and respiratory functions visibly improved and WBC count went back to a normal range.	first 5 minutes of first nebulisation were observed.	
Enilconazole										
Turkey (n=20)	Aerosol Spray	5mg/kg	5% solution	*Aspergillus flavus*	Lungs	NR	≈	Reduced lesion score in lungs (> 50%) but no effect in air sacs. Re-isolation rate of fungi: 80-100%	Body weights were significantly lower than non-infected turkeys.	[3]
Turkey (n=20)	PO	150mg/kg	NR	*Aspergillus flavus*	Lungs	NR	≈	Clear area of fungal growth inhibition seen in 30% of samples obtained from the turkeys but not when given via aerosol spray. Re-isolation rate of fungi: 80-100%	Body weights were significantly lower than non-infected turkeys.	[3]
Fluconazole										
African grey parrot (n=32)	PO	10-20mg/kg SD	Injectable, suspension of crushed tablets	NA	NA	NA	NA	Multiple-dosing studies on actual diseased birds are required to demonstrate efficacy.	Regurgitation (n=2) likely due to gavage administration or handling rather than drug toxicity as it was not observed in the multiple-dosing studies. Transient	[4]

									and mild increases in creatine kinase in a few birds likely due to muscle damage caused by handling. Increase in plasma bile acid concentrations (n=2) could possibly indicate hepatic dysfunction. Anemia (n=1) likely due to repeated blood samples taken.	
African grey parrot (n=15)	PO	10-20mg/kg q48h x 12 days	Suspension of crushed tablets	NA	NA	NA	NA	Findings in the study indicate that treatment with fluconazole administered PO 10 to 20 mg/kg every 24 to 48 hours is predicted to maintain plasma concentrations above the MIC (based on human isolates) of numerous strains of *Candida albicans* and *Candida tropicalis*. Studies on actual diseased birds are required to demonstrate efficacy.	No adverse effects were observed.	[4]
African grey parrot (n=13)	PO	15mg/kg q12h x 21-30 (mean) days	10mg/mL suspension	*Aspergillus spp.*	NR	NR	✓	Resolution of symptoms (n=11) and clinical improvement (n=2).	No adverse effects were observed.	[5]

African grey parrot (n=10)	PO	Fluconazole 15mg/kg q12h x 21-30 (mean) days + amphotericin B nebulisation (0.5mg/mL suspension)	10mg/mL suspension	*Aspergillus spp.*	NR	NR	✓	Resolution of symptoms (n=7) and clinical improvement (n=3).	Though 2 birds died, they were noted to be severely ill in their initial presentation making it unlikely for drug toxicity to be the cause of death. Side effects were only observed in 1 very old bird (40 y/o): weight loss, decreased activity, vomiting, dark stools and hypoproteinaemia possibly indicative of renal insufficiency due to cumulative effects with amphotericin B but inconclusive.	[5]
African grey parrot (n=1)	PO	15mg/kg q12-24h	NR	*Cryptococcus neoformans*	Respiratory tract, globe, periocular tissues, and brain	NR	×	Fluconazole was given together with a combination of other antifungals (terbinafine, then amphotericin B was added on, and both were dropped and replaced with flucytosine) but in general not	NR	[6]

								successful in managing the fungal infection.		
Citron-crested cockatoo (n=1)	PO	15mg/kg q12h	Suspension of grinded tablets	*Crypto-coccus gattii VGIIa*	Beak (and underlying bones), lungs, spleen and brain	MIC_{50} = 8	≈	Increased alertness, weight returned to normal and no new episodes of dyspnoea. However, 55 days after initial presentation, bird died acutely. Unclear if death is due to therapeutic inefficacy or severe disease.	NR	[7]
Cockatiel (n=28)	PO	10mg/kg SD	Grinded tablets in commercial suspending agent (Ora-plus)	NA	NA	NA	NA	AUC:MIC ≥ 25 is correlated with treatment success in humans treated for candidemia or mucosal candidiasis. Assuming linear kinetics and using MIC_{90} values from human fungal isolates, the predicted AUC:MIC for *C. albicans, C. krusei, C. glabrata, C. parapsilosis, C. tropicalis, C. lusitaniae, C. kefyr* were 298.56, 2.33, 4.66, 74.64, 74.64, 74.64 and 298.56 respectively. Hence, efficacy is likely to be achieved for susceptible Candida strains but multiple-dosing studies on actual diseased birds are required to demonstrate efficacy.	No adverse effects were observed.	[8]

Cockatiel (n=15)	PO	100mg/L x 8 days	Grinded tablets in drinking water	NA	NA	NA	NA	Efficacy is hard to determine due to small sample size and variabilities in individual water consumption which ultimately affects plasma fluconazole concentrations. Studies on actual diseased birds are required to demonstrate efficacy.	No adverse effects were observed.	[8]
Cockatiel (n=6)	PO	150mg/L x 8 days	Grinded tablets in drinking water	NA	NA	NA	NA	Efficacy is hard to determine due to small sample size and variabilities in individual water consumption which ultimately affects plasma fluconazole concentrations. Studies on actual diseased birds are required to demonstrate efficacy.	No adverse effects were observed.	[8]
Goldie's lorikeet (n=1)	PO	8mg/kg q24h x 30 days	NR	*Cryptococcus neoformans var gattii serovar B*	Upper beak and infraorbital sinus	64	×	No improvement in clinical symptoms and all strains were determined to be resistant to fluconazole.	NR	[9]
Papua lori (n=1)	Intralesional injection	8mg/kg q24h x 30 days	NR	*Cryptococcus neoformans var gattii serovar B*	Choana	64	×	Progressive increase in cryptococcal antigen titre and eventual fatal outcome. All strains were also determined to be resistant to fluconazole.	NR	[9]

Pesquet's parrot (n=1)	PO	10mg/kg BID	Tablets applied topically on food item	*Cryptococcus gattii*	Humerus	2.0	≈	Although clinically stable, failure to completely resolve existing lesions. Though plasma concentrations were above MIC, bone biopsy revealed persistent Cryptococcus growth. Therapeutic inefficacy likely due to low concentrations in bone tissues.	NR	[10]
Pesquet's parrot (n=1)	PO	15mg/kg q24h	NR	*Cryptococcus gattii*	Humerus, glottis, trachea	2.0	✓	Clinically stable, ability to fly improved, tracheal lesions resolved.	No adverse effects were observed.	[10]
Sun conure (n=1)	PO	5mg/kg q12h x 1 month	NR	*Candida albicans*	Respiratory tract	0.125	✓	Significant signs of improvement (increased appetite, normal body weight), clinically normal. Asymptomatic for 6 months since discontinuing all medications.	NR	[11]
Itraconazole										
African penguin (n=3)	PO	7mg/kg SD	Commercial formulation (Sporanox)	NA	NA	NA	NA	Multiple-dosing studies on actual diseased birds are required to demonstrate efficacy.	No adverse effects were observed.	[12]
African penguin (n=3)	PO	7mg/kg SD	Compounded formulation	NA	NA	NA	NA	Multiple-dosing studies on actual diseased birds are required to demonstrate efficacy.	No adverse effects were observed other than regurgitation (n=1).	[12]
African penguin (n=9)	PO	20mg/kg SD	Commercial formulation (Itrafungol)	NA	NA	NA	NA	Multiple-dosing studies on actual diseased birds are required to demonstrate efficacy.	No adverse effects were observed.	[13]

Blue-fronted Amazon parrot (n=8)	PO	5-10 mg/kg q24h x 14 days	Dissolved in HCL and orange juice	NA	NA	NA	≈	Predicted peak concentrations in plasma equalled or exceeded MIC for most (but not all) *Aspergillus* strains. The author also posits that a dose of 5mg/kg is unlikely to achieve therapeutic plasma concentrations against *Candida* strains.	No adverse effects were observed.	[14]
Peregrine falcon x Gyrfalcon hybrid (n=1)	PO	15mg/kg q12-24h x 3 months	Commercial formulation (Sporanox)	*Aspergillus spp.*	Upper and lower eyelids and top of the head	NR	✓	Lesions continued to spread to the whole head when given at a dose of 15mg/kg q24h. Resolution of lesions with normal skin was observed when frequency was increased to q12h in addition to a topical adjunct, miconazole, applied BID to the lesions. Feathers started to grow, but feather follicle cyst still present.	No adverse effects were observed.	[15]
Goliath heron (n=1)	PO	20mg/kg q12h x 50 days	Commercial formulation (Sporanox)	*Aspergillus fumigatus*	Pectoral muscle	NR	×	Initially clinical condition improved and wound reduced in size. However, despite adding topical miconazole twice weekly and topical povidone-iodine as adjuncts, no improvements were seen and *Aspergillus spp*. were still cultured.	NR	[16]

Humboldt penguin (n=17)	PO	6mg/kg q24h x 14days	Generic bulk compounded powder	NA	NA	NA	NA	NR	Significant but mild alterations in complete blood count and blood chemistry tests.	[17]
Humboldt penguin (n=6)	PO	6mg/kg q24h x 14days	Commercial capsules (Sporanox)	NA	NA	NA	NA	Susceptibility to Aspergillus fumigatus (MIC < 0.1µg/mL) is known to correlate well with C_{min} of 1µg/mL of the concentrations of both itraconazole and hydroxy-itraconazole combined. None of the birds exceeded this trough value.	Significant but mild alterations in complete blood count and blood chemistry tests.	[17]
Humboldt penguin (n=15)	PO	7mg/kg BID x 14 days	Commercial capsules (Sporanox)	NA	NA	NA	NA	Susceptibility to Aspergillus fumigatus (MIC < 0.1µg/mL) is known to correlate well with C_{min} of 1µg/mL of the concentrations of both itraconazole and hydroxy-itraconazole combined. Though trough values were achieved, they fell off before the end of the dosage interval. Based on the data, the author predicted that trough levels can be maintained throughout the entire duration of the dosing interval if a dose of 8.5mg/kg BID or 20mg/kg q24h is used. Studies on actual diseased birds	Significant but mild alterations in complete blood count and blood chemistry tests.	[17]

								are required to demonstrate efficacy.		
Humboldt penguin (n=11)	PO	12mg/kg q24h x 14days	Generic bulk compounded powder	NA	NA	NA	NA	NR	Significant but mild alterations in complete blood count and blood chemistry tests.	[17]
Humboldt penguin (n=4)	PO	12mg/kg q24h x 14days	Commercial capsules (Sporanox)	NA	NA	NA	NA	Susceptibility to Aspergillus fumigatus (MIC < 0.1μg/mL) is known to correlate well with C_{min} of 1μg/mL of the concentrations of both itraconazole and hydroxy-itraconazole combined. Though trough values were achieved, they fell off before the end of the dosage interval. Based on the data, the author predicted that trough levels can be maintained throughout the entire duration of the dosing interval if a dose of 8.5mg/kg BID or 20mg/kg q24h is used. Studies on actual diseased birds are required to demonstrate efficacy.	Significant but mild alterations in complete blood count and blood chemistry tests.	[17]
Japanese quail (n=60)	PO	10mg/kg q24h x 7 days	Sporex capsules administered in	*Aspergillus fumigatus*	Respiratory tract	NR	≈	Mortality rate reduced with 41/60 (68.3%) of the quails surviving compared to the untreated quails (none survived). No deaths	NR	[18]

			drinking water					occurred after 11 days post-inoculation.		
Japanese quail (n=18)	NEB	1% suspension SD x 30 min	1% nano-suspension (10% nano-suspension diluted with solution of 1.4% polysorbate 80)	NA	NA	NA	NA	High concentrations were reached in lungs and air sacs but studies on actual diseased birds are required to demonstrate efficacy.	No adverse effects were observed.	[19]
Japanese quail (n=18)	NEB	10% suspension SD x 30 mins	10% nano-suspension	NA	NA	NA	NA	High concentrations were reached in lungs and air sacs but studies on actual diseased birds are required to demonstrate efficacy.	No adverse effects were observed.	[19]
Japanese quail (n=18)	NEB	10% suspension for 30 min q24h x 5 days	10% nano-suspension	NA	NA	NA	NA	High concentrations were reached in lungs and air sacs but studies on actual diseased birds are required to demonstrate efficacy.	No adverse effects were observed.	[19]
Japanese quail (n=20)	NEB	4% suspension for 30 min q24h x 6 days	4% nano-suspension (10% nano-suspension diluted with solution of 1.4% polysorbate 80)	*Aspergillus fumigatus*	Trachea	0.125-0.5	≈	Retarded development of symptoms and lethality, but on day 7 following termination of treatment, 2 quails died.	No adverse effects were observed.	[20]
Japanese quail (n=20)	NEB	10% suspension for 30 min q24h x 6 days	10% nano-suspension	*Aspergillus fumigatus*	Trachea	0.125-0.5	✓	Blocked lethality and prevented disease-related symptoms in the low inoculation group and	No adverse effects were observed.	[20]

								retarded disease course in the high inoculation group.		
Japanese quail (n=6)	SC implant	24mg	NA	NA	NA	NA	×	Did not release sufficient drug to achieve targeted concentrations at any point during study.	No adverse effects were observed.	[21]
Lesser Flamingo (n=17)	PO	10mg/kg SD	Commercial solution (Itrafungol)	NA	NA	NA	≈	MIC_{90} of itraconazole (from another study) for avian *Aspergillus fumigatus* isolates was reported as 0.5 µg/mL. Plasma drug concentrations maintained above this MIC for at least 24h. Multiple-dosing studies on actual diseased birds are required to demonstrate efficacy.	No adverse effects were observed.	[22]
Lesser Flamingo (n=1)	PO	10mg/kg q24h x 10 days	Commercial formulation (Itrafungol)	*Candida albicans*	Gnathotheca, mandibular bone	NR	×	Continued losing weight, progressive weakness and eventually died.	NR	[23]
Mallard duck (n=15)	PO	20mg/kg SD	Acidified ITRA in HCL and orange juice	NA	NA	NA	NA	Studies on actual diseased birds are required to demonstrate efficacy.	No adverse effects were observed.	[24]
Mallard ducks (n=15)	PO	20mg/kg SD	ITRA in beta-cyclodextrin	NA	NA	NA	NA	Studies on actual diseased birds are required to demonstrate efficacy.	No adverse effects were observed.	[24]
Mallard ducks (n=4)	SC	250mg/kg	13.33% of polylactic-co-glycolic acid,	NA	NA	NA	×	Failed to achieve potentially therapeutic plasma concentrations.	Mild ataxia (n = 1) but unclear on which formulation.	[24]

			60% acetyl triethyl citrate, 6.7% triethyl citrate							
Mallard ducks (n=4)	SC	250mg/kg	13.33% of polylactic-co-glycolic acid, 60% triethyl citrate, 6.7% acetyl triethyl citrate	NA	NA	NA	×	Failed to achieve potentially therapeutic plasma concentrations.	Mild ataxia (n = 1) but unclear on which formulation.	[24]
Micronesian Kingfisher (n=1)	PO	20mg/kg BID	Commercial capsules	*Aspergillus spp.*	NR	NR	✓	Appetite, body weight and attitude returned to normal. Normal white blood count with resolved monocytosis. Lack of significant lesions were observed.	NR	[2]
Okinawa rail (n=1)	PO	10mg/kg BID x 162 days	NR	*Aspergillus flavus*	Left air sac	0.25	×	Respiratory distress did not improve and it eventually died.	NR	[25]
Pesquet's parrot (n=1)	PO	10mg/kg q24h	Compounded generic formulation	*Cryptococcus gattii*	Humerus	0.125	×	Developed dyspnea and dysphagia due to new *Cryptococcus* lesions in the proximal trachea and glottis. Plasma levels were undetectable.	NR	[10]
Red-tailed hawk (n=4)	PO	5mg/kg q24h x 15 days	Dissolved in HCL and orange juice	NA	NA	NA	NA	Plasma concentrations of both itraconazole and hydroxy-itraconazole were below 1.0 µg/mL which is used as a prognostic marker in humans. Studies on	No adverse effects were observed.	[26]

								actual diseased birds are required to demonstrate efficacy.		
Red-tailed hawk (n=7)	PO	10mg/kg q24h x 15 days	Dissolved in HCL and orange juice	NA	NA	NA	NA	Plasma concentrations of both itraconazole and hydroxy-itraconazole were below 1.0 µg/mL which is used as a prognostic marker in humans. Studies on actual diseased birds are required to demonstrate efficacy.	No adverse effects were observed.	[26]
Sun conure (n=1)	PO	10mg/kg q12h x 30 days	Commercial formulation	*Candida albicans*	Respiratory tract	0.03	≈	Improved clinically but repeated blood count showed increased leucocytosis with increased heterophilia and monocytosis.	NR	[11]
Turkey (n=20)	PO	6mg/kg	NR	*Aspergillus flavus*	Lungs	NR	≈	Significantly lower lesion score in lungs but not in air sacs & liver<br>Re-isolation rate of fungi: 60%	NR	[3]
Turkey (n=20)	PO	6mg/kg	NR	*Aspergillus flavus*	Lungs	NR	✓	Reduced lesion score in lungs and air sacs by more than 90%, with a mean lesion score of less than 0.2 in all organs.<br>Re-isolation rate of fungi: 30%	NR	[3]
Ketoconazole										
Lesser Sulphur-Crested Cockatoo (n=1)	PO	10mg/kg q12h x 3 weeks	NR	*Candida spp.*	Pro-ventriculus	NR	✓	Started eating normally, no relapses and appeared to be in excellent health.	NR	[27]

Turkey (n=40)	PO	50mg/kg	NR	*Aspergillus flavus*	Lungs	NR	≈	Reduced lesion score in lungs (> 50%) but no effect in air sacs. Re-isolation rate of fungi: 80-100%	NR	[3]
Voriconazole										
African grey parrot (n=12)	PO	6-18 mg/kg SD	In water or in suspending agents	NA	NA	NA	NA	Multiple-dosing studies on actual diseased birds are required to demonstrate efficacy. The author recommends a starting dose of 12mg/kg BID for aspergillosis based on the data obtained.	Mild weight loss and mildly high plasma creatinine kinase activity (n=12), mild transient increase in plasma aspartate aminotransferase activity (n=1)	[28]
African grey parrot (n=6)	PO	18mg/kg q12h x 9 days	NR	NR	NR	NR	NA	*Aspergillus spp.* known to have maximal fungicidal effect when concentrations are 3-4 times above the MIC. Trough values were however, < 0.4μg/mL (susceptible *Aspergillus spp.*). Studies on actual diseased birds are required to demonstrate efficacy.	Polyuria observed in all birds following first capture hence not likely due to drug toxicity, mild weight loss, mildly high creatine kinase, mildly high aspartate aminotransferase activity (n=1) (but clinically significance is unclear)	[28]
African grey parrot (n=1)	Topical	1 ophthalmic drop of 1% reconstituted solution OS q6h	Injectable solution reconstituted with	*Cryptococcus neoformans*	Eye	NA	≈	Discontinued after 5 days due to suspicion of systemic fungal invasion. Therapeutic success	Lethargy, ataxia, anorexia and loss of ability to perch 5 days upon	[6]

			buffered saline					undetermined due to short duration of therapy.	administration. Unclear if due to drug toxicity or systemic fungal invasion, indicating worsening disease.	
African penguin (n=18)	PO	5mg/kg SD	Suspension of tablets	NA	NA	NA	NA	Multiple-dosing studies on actual diseased birds are required to demonstrate efficacy.	Regurgitation (n=1).	[29]
African penguin (n=16)	PO	5mg/kg q24h x 8 days	Suspension of tablets	NA	NA	NA	NA	Therapeutic concentrations may not be achieved for invasive aspergillosis as the targeted therapeutic range of 2-6μg/mL is not achieved till days 4-7. Studies on actual diseased birds are required to demonstrate efficacy.	No adverse effects were observed.	[29]
African penguin (n=12)	PO	8.9-9.3mg/kg q12h and q24h<br><br>13.3mg/kg q12h<br><br>7.9-16.4mg/kg q24h	NA	NA	NA	NA	NA	NA	Anorexia (n=7), weakness (n=7), lethargy (n=7), ataxia (n=5), seizures/seizure-like activity (n=3), paraparesis (n=2), apparent change in vision (n=1)	[30]
Common raven (n=4)	PO	6mg/kg SD	Suspension of crushed tablets	NA	NA	NA	NA	The authors recommend that PO 6mg/kg given 2-3 times daily may be sufficient to reach target MIC	No adverse effects were observed.	[31]

								of 0.5-1µg/mL for Aspergillus spp. without reaching potentially toxic levels (≥ 5.0µg/mL). Multiple-dosing studies on actual diseased birds are required to demonstrate efficacy.		
Common raven (n=8)	PO	12mg/kg SD	Suspension of crushed tablets	NA	NA	NA	NA	Multiple-dosing studies on actual diseased birds are required to demonstrate efficacy.	No adverse effects were observed.	[31]
Common raven (n=4)	PO	24mg/kg SD	Suspension of crushed tablets	NA	NA	NA	NA	Multiple-dosing studies on actual diseased birds are required to demonstrate efficacy.	No adverse effects were observed.	[31]
Common raven (n=8)	IV	10mg/kg SD	Solution	NA	NA	NA	NA	The authors recommend that IV 10mg/kg given 2-3 times daily may be sufficient to reach target MIC of 0.5-1µg/mL for Aspergillus spp. without reaching potentially toxic levels (≥ 5.0µg/mL). Multiple-dosing studies on actual diseased birds are required to demonstrate efficacy.	No adverse effects were observed.	[31]
Emperor penguin (n=1)	PO	11.8mg/kg q72h	NA	NA	NA	NA	NA	NA	Anorexia, lethargy	[30]
Gentoo penguins (n=2)	PO	10.9-22.2mg/kg q24h	NA	NA	NA	NA	NA	NA	Lethargy, ataxia, change in mentation/depression	[30]

									(n=2). Weakness, ataxia (n=1)	
Gyrfalcons (n=1), Gyrfalcon x peregrine falcon hybrids (n=3), Saker falcon (n=3)	PO	12.5mg/kg q12h x 2 weeks	Solution of fine powder	NR	NR	1	NR	Median plasma concentration of 1.9-2.4 μg/mL was achieved within 1h of administration and peak values showed high interindividual variabilities with 22.7 μg/mL being the highest.	No adverse effects were observed. (1+2)	[32]
Gyrfalcons x peregrine falcon hybrid (n=1), Saker falcon (n=2)	PO	12.5mg/kg q12h x up to 91 days	Solution of fine powder	*Aspergillus spp.*	NR	1	NR	NR	No adverse effects were observed. (3)	[32]
Gyrfalcons (n=2), Saker falcons (n=3),	IM	12.5mg/kg SD	Suspension of powder	NA	NA	NA	NA	Plasma concentrations maintained above 1 μg/mL for 14.5-20h with an $AUC_{24unbound}$: MIC of 29.37 ± 4.12. $AUC_{24unbound}$: MIC ratio of 20-25 has been posited to be the best predictive measure of efficacy for *Candida albicans* and	No adverse effects were observed.	[33]

Peregrine falcons (n=2)								*Aspergillus fumigatus*. Dose of 12.5mg/kg once daily is thus likely to achieve therapeutic efficacy but multiple-dosing studies on actual diseased birds are required to demonstrate efficacy.		
Humboldt penguin (n=5)	PO	6.1-11.8mg/kg q12h<br><br>12.8-14.2mg/kg q24h	NA	NA	NA	NA	NA	NA	Anorexia (n=5), lethargy (n=4), change in mentation/depression (n=2), ataxia (n=2), weakness (n=1), seizures/seizure-like activity (n=1)	[30]
Japanese quail (n=38)	PO	20mg/kg SD	Commercial suspension	*Aspergillus spp.*	NA	NA	NA	Plasma concentrations peaked 2h post at 5.8 μg/mL and remained above 0.5 μg/mL for 4h.	No adverse effects were observed.	[34]
Japanese quail (n=10)	PO	20mg/kg q24h and euthanised on day 5 post infection	Commercial suspension	*Aspergillus fumigatus*	Trachea	4	✓	Only 1/7 had a positive culture for fungus.	No adverse effects were observed.	[34]
Japanese quail (n=10)	PO	20mg/kg q24h and euthanised on day 10 post infection	Commercial suspension	*Aspergillus fumigatus*	Trachea	4	≈	5/7 had detectable infection Statistically significant reduction in fungal burden between dose and IHC stained lung lobe sections.	No adverse effects were observed.	[34]

Japanese quail (n=38)	PO	40mg/kg SD	Commercial suspension	*Aspergillus spp.*	NA	NA	NA	Plasma concentrations peaked 2h post dose at 6.9 µg/mL and remained above 0.5 µg/mL for 12h.	No adverse effects were observed.	[34]
Japanese quail (n=10)	PO (by gavage)	40mg/kg q24h and euthanised on day 5 post infection	Commercial suspension	*Aspergillus fumigatus*	Trachea	4	≈	All birds survived and 9/10 had demonstrable infection in lungs. Significant reduction in colony-forming units as compared to diluent-treated birds.	No adverse effects were observed.	[34]
Japanese quail (n=10)	PO (by gavage)	40mg/kg q24h and euthanised on day 10 post infection	Commercial suspension	*Aspergillus fumigatus*	Trachea	4	≈	8/9 had residual infection Significant reduction in acute inflammation in comparison to diluent-treated birds.	No adverse effects were observed.	[34]
Japanese quail (n=6)	SC implant	24mg	NA	NA	NA	NA	×	Did not release sufficient drug to achieve targeted concentrations at any point during study.	No adverse effects were observed.	[21]
Khaki Campbell duck (n=1)	PO	20mg/kg q12h x 35 days	NR	*Aspergillus fumigatus*	Eyes	NR	✓	Increased weight, appetite and energy level returned to normal. Both eyes were open and appeared comfortable with continuous improvement through clearing of fibrosis at dorsal and dorsomedial cornea, increased corneal vascularisation and return of pupillary light response.	Mild leucocytosis with moderate monocytosis was observed.	[35]
Khaki Campbell duck (n=1)	Topical	1 drop OS q4-6h	Suspension	*Aspergillus fumigatus*	Eyes	NR	≈	Layer of plaque overlying cornea had fallen off. Appetite decreased. Blepharospasm remained unchanged although blepharedema,	NR	[35]

								conjunctival hyperemia and chemosis showed improvement.		
Macaroni penguin (n=1)	PO	10.2mg/kg q24h	NA	NA	NA	NA	NA	NA	Ataxia, paresis, apparent change in vision, seizures/seizure-like activity	[30]
Magellanic penguin (n=15)	PO	2.5mg/kg SD	Suspension of powder	NA	NA	NA	NA	Multiple-dosing studies on actual diseased birds are required to demonstrate efficacy.	No adverse effects were observed.	[36]
Magellanic penguin (n=30)	PO	5mg/kg SD	Suspension of powder	NA	NA	NA	NA	Multiple-dosing studies on actual diseased birds are required to demonstrate efficacy.	No adverse effects were observed.	[36]
Magellanic penguin (n=1)	PO	8mg/kg SID → 5mg/kg SID → 5 days on-2-day off regimen x several years	Suspension of crushed tablets	*Aspergillus fumigatus*	NA	NA	≈	Apparent increase in weight.	Anorexia, no further episodes of toxicity when given via 5 day on-2 day off regimen	[36]
Magellanic penguin (n=3)	PO	8.1mg/kg q24h then q48h then 12.2mg/kg q72h<br><br>8.5mg/kg q24h<br><br>14.7mg/kg q12h, 5 days on 2 days off	NA	NA	NA	NA	NA	NA	Anorexia (n=3), change in mentation/depression (n=3), lethargy (n=2), ataxia (n=1), apparent change in vision (n=1), seizures/seizure-like activity (n=1)	[30]

Mallard duck (n=2)	PO	10mg/kg SD	Suspension	NA	NA	NA	NA	Plasma concentration above targeted MIC of 0.5 μg/mL for minimum of 5.2h. Multiple-dosing studies on actual diseased birds are required to demonstrate efficacy.	No adverse effects were observed.	[37]
Mallard duck (n=6)	PO	10mg/kg SD	Solution	NA	NA	NA	NA	Multiple-dosing studies on actual diseased birds are required to demonstrate efficacy.	No adverse effects were observed.	[37]
Mallard duck (n=2)	PO	20mg/kg SD	Suspension	NA	NA	NA	NA	Closest to maintaining plasma concentration (among SD studies) at 0.5 μg/mL without exceeding trough Cmax of 5.5 μg/mL. Multiple-dosing studies on actual diseased birds are required to demonstrate efficacy.	No adverse effects were observed.	[37]
Mallard duck (n=3)	PO	40mg/kg SD	Suspension	NA	NA	NA	NA	Plasma concentration above 0.5 μg/mL for maximum of 12h. Multiple-dosing studies on actual diseased birds are required to demonstrate efficacy.	No adverse effects were observed.	[37]
Mallard duck (n=18)	PO	20mg/kg SID x 21 days	Suspension	NA	NA	NA	×	Plasma concentrations were not sustained above the targeted MIC of 0.5 μg/mL for the entire dosing interval. The author recommends a shorter dosing interval q8h or q12h at the same dose but more studies on actual diseased birds	No adverse effects were observed.	[37]

								are required to demonstrate efficacy.		
Mallard duck (n=6)	IV	10mg/kg SD	Solution	NA	NA	NA	NA	Multiple-dosing studies on actual diseased birds are required to demonstrate efficacy.	No adverse effects were observed.	[37]
Okinawa rail (n=1)	IM	23.5mg/kg SID/BID x 162 days	NR	*Aspergillus flavus*	Left air sac	2	×	Respiratory distress did not improve and it eventually died.	NR	[25]
Racing pigeon (n=10)	PO	10mg/kg q12h x 14 days	Suspension of crushed tablets	*Aspergillus fumigatus*	Apical part of right lung	0.25	✓	Eliminated fungus in the near absence of macroscopic liver pathology. Showed significantly less severe clinical signs compared to 20mg/kg q24h group.	Mild histological liver abnormalities. Moderate oval cell proliferation of limiting plate and moderate bile duct proliferation in 3/6 pigeons which were postulated to be a sign of regeneration as a result of hepatocellular degeneration.	[38]
Racing pigeon (n=10)	PO	20mg/kg q24h x 14 days	Suspension of crushed tablets	*Aspergillus fumigatus*	Apical part of right lung	0.25	✓	Reduced fungi isolation rates. Significant reduction of aspergillosis-associated clinical signs and lesions compared to sham-treated animals.	Mild histological and macroscopic liver abnormalities.	[38]
Racing pigeon (n=16)	IM	12.5mg/kg BID x 7 days	Reconstituted with sterile water	NR	NR	NR	NA	NR	Significant uric acid increase and phosphorus decrease (but	[39]

									still within reference range). Inflammation in the pectoral muscle predominantly on the voriconazole-injected sites.	
Red-tailed hawk (n=12)	PO	10mg/kg SD	Suspension	NR	NA	NA	NA	Duration that drug concentrations remained above targeted MIC_{90} of 1 μg/mL was 8.8 ± 1.1h. Multiple-dosing studies on actual diseased birds are required to demonstrate efficacy.	No adverse effects were observed.	[40]
Red-tailed hawk (n=8)	PO	10mg/kg q12h x 14 days	NR	NR	NR	NR	≈	Duration that drug concentrations remained above targeted MIC_{90} of 1 μg/mL was 6.4 ± 1.5h where the mean plasma concentrations were 4.5 ± 2.7 μg/mL. Studies on actual diseased birds are required to demonstrate efficacy and determine the T > MIC for red-tailed hawks.	No adverse effects were observed.	[40]
Red-tailed hawk (n=7)	PO	15mg/kg SD	Suspension of powder	NA	NA	NA	NA	NA	No adverse effects were observed.	[41]
**ALLYLAMINES**										
Terbinafine										

Common shelduck (n=7)	PO	60mg/kg SD	Suspension of tablet	NA	NA	NA	NA	Plasma concentrations maintained above mentioned fungal species for almost 6h, and very close to 1 μg/mL at 8h sampling time. Peak levels reached above highest minimum fungicidal concentration for *Aspergillus fumigatus* (4μg/mL) for at least 1h and almost 8h for *Aspergillus flavus*. Multiple-dosing studies on actual diseased birds are required to demonstrate efficacy.	No adverse effects were observed.	[42]
Hispaniolan Amazon parrot (n=6)	PO	60mg/kg SD	Suspension of tablets	NA	NA	NA	NA	Peak concentrations within range of in vitro MIC but failed to reach therapeutic range of 1-4 μg/mL suggested for red-tailed hawks. Multiple-dosing studies on actual diseased birds are required to demonstrate efficacy.	No adverse effects were observed.	[43]
Japanese quail (n=6)	SC implant	24mg	NA	NA	NA	NA	≈	Target concentrations reached at some time points but inconsistent.	No adverse effects were observed.	[21]
**POLYENES**										
Amphotericin B										
African grey parrots (n=1)	IV catheter	1.5mg/kg	NR	*Cryptococcus neoformans*	Respiratory tract, globe, periocular	NR	×	Bird became more responsive and was observed eating that night. Shortly after recovery, the bird experienced seizures and died.	NR	[6]

					tissues, and brain					
Goliath heron (n=1)	Topical	1.35mg/kg q24h x 30 days → discontinued for 15 days → q24h x 14 days → every 3/4 days x 16 days → every 5-7 days x 60 days	Liposomally encapsulated with gelatin lubricant	*Aspergillus fumigatus*	Pectoral muscle	NR	✓	Discharge greatly diminished and general appearance of wound greatly improved. Follow-up fungal culture was negative.	No adverse effects were observed.	[16]
Gyrfalcon (n=1)	Intra-tracheal	1mg/kg BID x 3 days	NR	*Aspergillus spp.*	Respiratory tract	NR	≈	Stabilisation of condition but only dramatically improved upon clotrimazole nebulisation.	No adverse effects were observed.	[1]
Gyrfalcon (n=1)	IV	1.5mg/kg TID x 3 days	NR	*Aspergillus spp.*	Respiratory tract	NR	≈	Stabilisation of condition but only dramatically improved upon clotrimazole nebulisation.	No adverse effects were observed.	[1]
Gyrfalcon (n=1)	NEB	1mg/mL of BID x 20 days	Saline solution	*Aspergillus spp.*	Respiratory tract	NR	≈	Stabilisation of condition but only dramatically improved upon clotrimazole nebulisation.	No adverse effects were observed.	[1]
Peruvian penguin (n=1)	IM	2.0mg/kg SID	NR	*Aspergillus fumigatus*	Respiratory tract, air sac	NR	×	Became sick and succumbed to disease.	NR	[44]
Peruvian penguin (n=1)	Intra-peritoneal	NR	Aminoplex	*Aspergillus fumigatus*	Respiratory tract, air sac	NR	×	Became sick and succumbed to disease.	NR	[44]
Prairie falcon (n=2)	IV	0.5-0.75 mg	Fungizone	*Aspergillus spp*	Respiratory tract	NR	×	Died despite IV administration and initiation of oxygen therapy.	NR	[45]

Sun conure (n=1)	Intralesional	1mg/kg	NR	*Candia albicans*	Respiratory tract	NR	≈	Uneventful recovery	NR	[11]
Sun conure (n=1)	NEB	1mg/mL for 15 mins q8h x 2 days	Saline solution	*Candida albicans*	Respiratory tract	NR	≈	Improved clinically but repeated blood count showed increased leucocytosis with increased heterophilia & monocytosis.	NR	[11]
Yellow-naped Amazon (n=1)	NEB	1mg/mL BID x 31 days	Saline solution	*Aspergillus spp.*	Respiratory tract	NR	≈	Dramatic response to therapy but still required clotrimazole nebulisation subsequently for clinical improvement.	No adverse effects were observed.	[1]
Barrow's golden-eye, common scoter, red-breasted merganser, long-tailed duck (n=NR)	Topical	Once daily	4% ointment	*Candida albicans*	Membrane nictitans, cornea	NR	✓	Effective in majority of cases provided birds kept out of water for at least 30 minutes after application, if continued for 14 days or more, provided infection not within globe.	NR	[46]
Barrow's golden-eye, common scoter, red-breasted	PO	NR	NR	*Candida albicans*	Membrane nictitans, cornea	NR	NR	NR	Not well tolerated by ducks and led to digestive upsets & diarrhoea.	[46]

merganser, long-tailed duck (n=NR)										
Great-horned owl (n=5), red-tailed hawk (n=5)	IV	3 doses at rate of 1.5mg/kg at 2h intervals	NR	NR	NR	NR	NR	Detectable levels found at 6h after last injection. No detectable concentrations at 24h post-IV administration.	Transitory episode of incoordination and mild convulsions, which passed in < 1 minute.	[47]
Nystatin										
Sun conure (n=1)	PO	400,000 units/kg q12h	NR	*Candida albicans*	Pulmonary candidiasis	NR	≈	Mild clinical improvement.	NR	[11]
**NUCLEOSIDE ANALOGUES**										
Flucytosine										
Crested wood partridge (n=4)	PO	60mg/kg BID	Commercial formulation (Ancoban)	*Aspergillus spp.*	Respiratory tract	NR	×	Yellow plaques along surfaces of thoracic air sacs, & yellow nodules over surface & throughout parenchyma of lungs. Lungs contained areas of haemorrhage.	NR	[48]
Gyrfalcon (n=1)	PO	120mg/kg QID x 20 days	NR	*Aspergillus spp.*	Respiratory tract	NR	✓	Stabilisation of condition.	No adverse effects were observed.	[1]
Pesquet's parrot (n=1)	PO	50mg/kg q12h	Compounded suspension	*Cryptococcus gattii*	Humerus	NR	×	Plasma drug concentrations were undetectable.	NR	[10]

Barrow's golden-eye, common scoter, red-breasted merganser, long-tailed duck (n=NR)	Parenteral	400mg/kg/24h given as 2 divided doses over a period of 3 weeks	Commercial formulation (Alcobon)	*Candida albicans*	Membrane nictitans, cornea	NR	✓	Completely cleared intraocular infection after 12-14 days.	No adverse effects were observed.	[46]
Red-tailed hawk (n=5), Great-horned owl (n=5)	PO	3 doses over 24h totalling 120mg/kg	NR	NR	NR	NR	NR	Fungal inhibitory concentrations were achieved only at 2 and 6h post-administration, but not at 24h post-administration.	NR	[47]
**ECHINOCANDINS**										
Micafungin										
Okinawa rail (n=1)	SC	11.6 or 22.7 mg/kg SID/BID x 162 days	NR	*Aspergillus flavus*	Left air sac	>32	×	Respiratory stress did not improve and it eventually died.	NR	[25]

$AUC_{24unbound}$:MIC = ratio of 24-hour area under concentration-time curve versus minimum inhibitory concentration; **BID** = twice daily; **TID** = thrice daily; **C_{max}** = peak plasma drug concentration; **C_{min}** = trough plasma drug concentration; **IM** = intramuscular; **IV** = intravenous; **MIC** = minimum inhibitory concentration; **MIC_{50}**, **MIC_{90}** = lowest concentration of the antifungal at which 50% and 90% of the isolates were inhibited, respectively; **n** = number of animals; **NEB** = nebulization; **NA** = not applicable; **NR** = not reported; **OS** = left eye; **PEG** = polyethylene glycol; **PO** = oral; **QID** = four times daily; **ROA** = route of administration; **SC** = subcutaneous; **SD** = single dose; **SID** = once daily; **SOI** = site of infection; **WBC** = white blood cell; ✓ = efficacy observed; ≈ = possible sign of efficacy but inconclusive; × = no efficacy observed.

Table S2. Efficacy and safety parameters of commonly reported antifungals for reptile species.

Species	ROA	Dosing regimen	Causative pathogen	SOI	MIC (μg/mL)	Efficacy	Treatment outcomes / Measures of efficacy	Adverse effects	Ref.
**AZOLES**									
Fluconazole									
Galapagos tortoise (n=1)	PO	2mg/kg SD	*Exophiala pisciphila*	Systemic	-	×	Clinical condition deteriorated with periodic regurgitation upon feeding. Tortoise became moribund 1 week later with tachypnea, dyspnea, tachycardia, a holosystolic heart murmur, and a friction rub. and subsequently euthanised due to poor prognosis. Lesions were in concordance with disseminated phaeohyphomycosis especially in the lungs and eyes.	No adverse effects were observed. Symptoms prior to death most likely attributed to the deteriorating clinical condition rather than drug toxicity.	[49]
Kemp's Ridley sea turtle (n=21)	SC	21 mg/kg loading dose, followed by 10 mg/kg q5d		-	> 64 μg/mL for 2 turtles that died	≈	Most of the turtles (n=19) were successfully rehabilitated and released to the wild. However, efficacy of fluconazole is unclear due to marked variabilities in disease conditions and concurrent bacterial infection, which was treated with ceftazidime. The2 turtles that died had fungal pneumonia and pleuritis, caused by *Beauvaria bassiana sp.*, as well as necro- ulcerative enteritis. Causative pathogens were resistant to fluconazole.	NR	[50]

Logger-head sea turtle (n=6)	IV	2.5mg/kg q24h x 5 days	NA	-	-	NA	NR	No adverse effects were observed.	[51]
Logger-head sea turtle (n=6)	SC	2.5mg/kg q24h x 5 days	NA	-	-	NA	Therapeutic plasma concentrations of 8 µg/mL were likely to be reached in around 30 days because of its prolonged half-life. Longer multiple-dosing studies on actual diseased turtles are required to demonstrate efficacy.	No adverse effects were observed.	[51]
Logger-head sea turtle (n=4)	SC	21 mg/kg loading dose, followed by 10 mg/kg q5d	NA	-	-	≈	With the multiple-dose regimen, plasma concentrations ranged from around 8-19 µg/mL which has been reported to be effective against *Aspergillus spp.* in sea turtles and other reptiles. More studies on actual diseased turtles are required to demonstrate efficacy.	No adverse effects were observed.	[51]
Itraconazole									
Boa constrictor (n=1)	PO	5mg/kg itraconazole q24h + 1% silver sulfadiazine cream topically q12h	CANV	Skin	-	×	Snake died after 3 weeks of antifungal treatment.	NR	[52]
Coastal bearded dragon (n=1)	PO	5mg/kg q24h	CANV	Skin	-	×	Euthanised due to persistent systemic mycoses with several granulomas indicating the presence of fungal infections.	NR	[53]
Coastal bearded	PO	10mg/kg q24h	*NR*	Skin	-	×	Multifocal hepatic granulomas with the presence of numerous septate hyphae	NR	[54]

dragon (n=1)							were found upon biopsy. Fungal culture of lesions revealed fungal pathogens of morphological characteristics inconsistent from the original suspicion of *Paranannizziopsis australasiensis*.		
Corn snake (n=1)	PO	0.7mg q62h x 155 days	*Aspergillus fumigatus*	Cuta-ne-ous/ deep SC	-	✓	Metabolically scaled doses were used. Successful treatment with follow-up showing no recrudescence of infection.	No adverse effects were observed.	[55]
Cotton-mouth (n=7)	Per cloaca	10mg/kg SD	*Ophidiomyces ophiodiicola*	Skin	1.0	NA	Therapeutic concentrations of itraconazole and hydroxyitraconazole in plasma and tissues were not reached. Therapeutic efficacy unlikely to be observed clinically. Multiple-dosing studies on actual diseased snakes are required to demonstrate efficacy.	No adverse effects were observed.	[56]
Fly river turtle (n=3)	PO	10 mg/kg q48h x 20 days + 0.038% malachite green and 4.26% formaldehyde dips for 15 min q12h x 33 days	*Paecilomyces lilacinus*	Systemic	-	✓	By day 57, all the lesions had regressed and malachite green, formaldehyde dips, and itraconazole treatment were discontinued. By day 104, the turtles were active and eating well and no other lesions were present.	No adverse effects were observed.	[57]
Garter snake (n=1)	PO	0.4mg q50h x 162 days	*Aspergillus niger*	Cuta-ne-ous/ deep SC	-	✓	Metabolically scaled doses were used. Successful treatment with follow-up showing no recrudescence of infection.	No adverse effects were observed.	[55]

Garter snake (n=1)	PO	0.5mg q53h x 184 days	*Aspergillus niger*	Cuta-ne-ous/ deep SC	-	✓	Metabolically scaled doses were used. Successful treatment with follow-up showing no recrudescence of infection.	No adverse effects were observed.	[55]
Green iguana (n=1)	PO	1.2mg q75h x 159 days	*Aspergillus fumigatus*	Cuta-ne-ous/ deep SC	-	✓	Metabolically scaled doses were used. Successful treatment with follow-up showing no recrudescence of infection.	No adverse effects were observed.	[55]
Green iguana (n=1)	PO	1.7mg q83h x 184 days	*Aspergillus fumigatus*	Cuta-ne-ous/ deep SC	-	✓	Metabolically scaled doses were used. Successful treatment with follow-up showing no recrudescence of infection.	No adverse effects were observed.	[55]
Inland bearded dragon (n=1)	PO	0.8mg q65h x 155 days	*Aspergillus fumigatus*	Cuta-ne-ous/ deep SC	-	✓	Metabolically scaled doses were used. Successful treatment with follow-up showing no recrudescence of infection.	No adverse effects were observed.	[55]
Inland bearded dragon (n=1)	PO	0.8mg q63h x 148 days	*Aspergillus fumigatus*	Cuta-ne-ous/ deep SC	-	✓	Metabolically scaled doses were used. Successful treatment with follow-up showing no recrudescence of infection.	No adverse effects were observed.	[55]
Inland bearded dragon (n=7)	PO	5mg/kg q24h	CANV	Skin	-	≈	Only adequately effective since after ap-proximately 4 weeks, the fungus was not re-isolated from the dermal lesions.	5/7 of the bearded drag-ons died and CANV was isolated from the skin of 2 of these lizards. Significantly increased levels of AST was also observed in 4 bearded dragons which could be suggestive of toxicity.	[58]
Inland bearded	PO	5mg/kg q48h	CANV	Skin	-	✓	Surgical site was healed 2 weeks later with some weight gain. After 6 months	NR	[59]

dragon (n=1)		x 14 days + 20mg/kg SC ceftazidime q72h x 12 days					from initial presentation, the lizard remained free of dermal lesions.		
Inland bearded dragon (n=1)	PO	10mg/kg q24h x 6 weeks + 0.125% chlorhexidine topical solution	CANV	Skin	-	×	After 3 weeks, the maxillary lesion had reduced in size. 6 months upon discontinuation, the lesion recurred beyond the margins of the original lesion. CANV was cultured and despite re-initiation of itraconazole therapy with topical miconazole, there was only mild improvement. Treatment was subsequently discontinued and supportive care was initiated. The lizard died 48h later.	Post-mortem examination revealed a diffuse hepatopathy which could be suggestive of itraconazole toxicity.	[59]
Inland bearded dragon (n=1)	PO	10mg/kg q24h x 10 weeks + daily baths in dilute povidone-iodine solution	CANV	Skin	-	×	Dermatitis improved initially for 8 weeks, but the bearded dragon continued to present with anorexia and significant weight loss, and was euthanised 2 weeks later. Post-mortem examination revealed an extensive ulcerative dermatitis of the ventral abdomen and a focal hepatic granuloma. Bacterial rods and fungal hyphae were found within the granuloma, indicating progressive disease despite itraconazole treatment.	NR	[59]

Jewel chameleon (n=1)	PO	10mg/kg q24h x 21 days	CANV	Systemic	-	×	6 days after initiation of therapy, the chameleon died. Numerous fungal hyphae were found in the granulomas in lung and kidney tissues.	No adverse effects were observed.	[60]
Kemp's Ridley sea turtle (n=3)	PO	5mg/kg q24h x at least 30 days	NA	-	-	≈	Plasma concentrations had an effective trough concentration of 0.5 µg/mL. MIC of *Candida spp.* and *Cryptococcus neoformans* ≤ 0.12 µg/mL considered to be susceptible; if MIC = 0.25-0.50 µg/mL then susceptibility depends on the dose administered.	No adverse effects were observed.	[61]
Kemp's Ridley sea turtle (n=1)	PO	5mg/kg q24h	*Colletotrichum acutatum*	Systemic	-	×	Prophylactic treatment with itraconazole did not resolve the fungal infection. Lesions from various tissues revealed multicentric systemic fungal disease. Efficacy of itraconazole is inconclusive due to concurrent use of antibiotics and severe disease of the turtle.	Kidney failure and likely hepatic compromise were observed with high numbers of toxic heterophils. Turtle showed clinical deterioration and subsequently died. Inconclusive on whether this was due to severe illness or drug toxicity.	[62]
Kemp's Ridley sea turtle (n=3)	PO	15mg/kg q72h x at least 30 days	NA	-	-	≈	Plasma concentrations had an effective trough concentration of 0.5 µg/mL. MIC of *Candida spp.* and *Cryptococcus neoformans* ≤ 0.12 µg/mL considered to be susceptible; if MIC = 0.25-0.50 µg/mL then	No adverse effects were observed.	[61]

							susceptibility depends on the dose administered.		
Panther chameleon (n=1)	PO	5mg/kg q24h	*Aspergillus spp.*	Bone	-	≈	Upon follow-up at 2 weeks, more than half of the tongue sloughed and 6 weeks later, it regurgitated after administration of itraconazole, which led to discontinuation. Chameleon was reported to be well despite discontinuation of antimicrobials.	NR	[63]
Parson's chameleon (n=1)	PO	10mg/kg q24h x 21 days	CANV	Skin	-	≈	General loss of condition as the chameleon appeared to favour the left hind leg and would not grasp perches as easily as it did before. Chameleon's appetite and overall condition improved months later but the chameleon still died a year later. Autopsy indicated that death was due to cholecystitis and septicaemia and no evidence was mycosis was seen.	Radiographs revealed hepatomegaly which could be a result of possible itraconazole toxicity. Elevations in serum creatinine kinase and AST activities were also noted.	[60]
Spiny lizard (n=35)	PO	23.5mg/kg (mean) q24h x 3 days	NA	Systemic	-	NA	Reported that the itraconazole plasma and liver concentrations were above the MICs of numerous fungal pathogens (*Aspergillus spp.*: 0.063-0.13, *Candida spp.*: 0.063-128, *Trichophyton spp.*: 0.063-64, *Microsporum spp.*: 0.063-0.25, *Zygomycetes*: 0.063- > 128 µg/mL) for 6 days beyond the peak concentration. Longer multiple-dosing	NR	[64]

							studies on actual diseased lizards are required to demonstrate efficacy.		
Tuatara (n=1)	PO	5mg/kg q24h x 28 days + topical 1% terbinafine ointment x 21 days	*Paranan-nizziopsis australasiensis*	Skin	-	≈	Lesions healed and with ecdysis, skin eventually returned to normal. Subsequently, new lesions developed and fungal culture revealed fungus that was morphologically similar to *Parananniz-ziopsis australasiensis.*	NR	[54]
Tuatara (n=1)	PO	5mg/kg q24h x 29 days	*Paranan-nizziopsis australasiensis*	Skin	-	✓	Lesions resolved after 29 days, resulting in discontinuation of therapy.	NR	[54]
Ketoconazole									
Black ratsnake (n=1)	PO	50mg/kg q24h	*Chryso-sporium ophiodiicola*	Mandible and eye	-	×	Postoperative swelling reduced but the snake passed away 2 months post-surgery.	NR	[65]
Gopher tortoise (n=14)	PO	30mg/kg q24h	NA	-	-	≈	Therapeutic concentrations (> 1 μg/mL) were maintained during time intervals from 4 to 32h after oral dosing.	Rise in AST and LDH were observed and reported to be most likely due to immobilisation with succinylcholine and procedures for catheter placement rather than drug toxicity.	[66]
Gopher tortoise (n=8)	PO	15mg/kg q24h	NA	Superficial and deep mycoses	-	≈	The author quoted that plasma concentrations of 1-4μg/mL were 81% effective against superficial and deep mycoses. Trough concentration of 1.2 μg/mL maintained @ steady-state (> target MIC	No adverse events were observed other than insignificant changes in serum biochemistry.	[67]

							of 1 μg/mL). More studies on actual diseased turtles are required to demonstrate efficacy.		
Gopher tortoise (n=8)	PO	30mg/kg q24h	NA	Superficial and deep mycoses	-	≈	The author quoted that plasma concentrations of 1-4μg/mL were 81% effective against superficial and deep mycoses. Trough concentration of 2.4 μg/mL maintained @ steady-state (> target MIC of 1 μg/mL). More studies on actual diseased turtles are required to demonstrate efficacy.	No adverse events were observed other than insignificant changes in serum biochemistry.	[67]
Green Iguana (n=1)	PO	20mg/kg q24h + 2% chlorhexidine solution + topical terbinafine	*Chrysosporium spp.*	Skin	KTZ[a]: 50-54mm ITZ[a]: 30-32mm TBF[a]: 78-80mm	✓	Re-evaluated after 1 month and new healthy skin was developing at the site of previous lesion, which improved with ecdysis.	NR	[68]
Green Iguana (n=1)	PO	20mg/kg q24h x 14 weeks + 2% chlorhexidine solution + topical terbinafine	*Chrysosporium spp.*	Skin	KTZ[a]: 50-54mm ITZ[a]: 30-32mm TBF[a]: 78-80mm	✓	5 months after initial presentation, the lesions had completely regressed.	NR	[68]
Jackson's chameleon (n=1)	PO	25mg/kg q2d x 4 weeks	*Trichophyton sp.*	Skin	-	×	Did not significantly alter the progression of the lesions.	NR	[60]
Voriconazole									

Cotton-mouth(n=6)	SC	5mg/kg SD	*Ophidiomyces ophiodiicola*	Skin	0.25	NA	In the surviving 2 cottonmouths, plasma concentrations were above the MIC for the first 12 and 24h after administration respectively. Multiple-dosing studies on actual diseased snakes are required to demonstrate efficacy.	Only 2 survived with no adverse effects noted. The other 4 cotton-mouths died and presented with lethargy, depression, loss of righting reflex. Torticollis was also observed in 1 of the cottonmouths.	[56]
Cotton-mouth (n=1)	SC	10mg/kg SD	*Ophidiomyces ophiodiicola*	Skin	0.25	NA	Plasma concentrations were above the MIC for the first 12 after administration. Multiple-dosing studies on actual diseased snakes are required to demonstrate efficacy.	No adverse effects were observed.	[56]
Eastern massa-sauga (n=2)	SC Osmotic pump	22.2 mg/mL (1.02-1.6 mg/kg/h)	*Ophidiomyces ophiodiicola*	Skin	0.25	×	Therapeutic plasma concentrations were not reached and severe granulomatous and heterophilic sinusitis and pleuro-pneumonia surrounded by fungal hyphae were found. However, sample size is too small to make any generalised conclusions on efficacy.	NR	[56]
Giant girdled lizard (n=1)	PO	10mg/kg q24h	CANV	Skin	AMB: 1<br>ITZ: 0.5<br>VOR: 0.25<br>TBF:0.5	✓	CANV isolates were cleared.	No adverse effects were observed.	[69]
Guthega skink (n=4)	PO	10mg/kg q24h + shallow baths of benzalkonium	*Lecanicillium spp*	Skin	-	✓	Clinical signs of disease were resolved.	NR	[70]

		chloride and polyhexamethylene biguanide HCL (1 : 250 dilution for 20 min q24h)							
Inland bearded dragon (n=7)	PO	10 mg/kg q24h	CANV	Skin	-	✓	CANV infection was cleared in all 6 voriconazole-treated animals that survived.	1/7 died after 25 days of treatment and CANV was isolated from the liver and lungs. Significantly increased levels of AST was also observed in 3 bearded dragons which could be suggestive of drug toxicity.	[58]
Northwestern pond turtle (n=6)	SC	10 mg/kg q48h x 14 days	*Emydomyces testavorans*	Shell	FLU: 1<br>ITRA: 0.03<br>POS: 0.03<br>TBF: 0.03<br>VOR: 0.06	≈	Observed trough plasma concentrations were above the reported MICs for *E. testavorans*.	No other adverse events were observed other than a significant increase in the polychromatophilic cells.	[71]
Red-eared slider turtle (n=12)	SC	10 mg/kg q12h x 7 days	NA	Systemic	-	×	Likely to have therapeutic efficacy only when voriconazole MIC is ≤0.125 μg/mL based on published MIC data for reptilian fungal isolates according to the author.	2 turtles showed signs of lower respiratory tract disease and hind limb paresis and were eventually euthanized.	[72]

Red-eared slider turtle (n=10)	SC	10 mg/kg q12h x 7 days	NA	Systemic	-	NA	No blood samples were collected to quantify plasma concentrations as this was a safety study.	No adverse effects were observed except for mild-to-moderate injection site skin inflammation in 8 of the turtles which were likely due to repeated administrations. Slight increases in absolute and relative monocyte counts were also noted.	[72]
Timber rattlesnake (n=1)	SC Osmotic pumps	10 mg/mL (12.1–17.5 mg/kg/h)	*Ophidiomyces ophiodiicola*	Skin	0.25	≈	Therapeutic plasma concentrations were reached (> 0.25 µg/mL) approximately 2 weeks after initial pump placement and remained above therapeutic levels during all 3-months of treatment. Therapeutic efficacy cannot be concluded due to small sample size.	NR	[56]
**ALLYLAMINES**									
Terbinafine									
Cottonmouth (n=7)	NEB	2 mg/mL (18 mg total dose) x 30 min	*Ophidiomyces ophiodiicola*	Skin	-	≈	In vitro susceptibility concentration (0.015 µg/mL) was maintained for at least 12h in 4 of the nebulized cottonmouths.	No adverse effects were observed but all snakes non-significantly gained weight.	[73]
Cottonmouth (n=7)	SC implant	24.5 mg (75–190 mg/kg)	*Ophidiomyces ophiodiicola*	Skin	-	≈	In vitro susceptibility concentration (0.015 µg/mL) was maintained for > 5 weeks in implanted snakes (peak concentration of 100 ng/mL at 3 weeks).	No adverse effects were observed but all snakes non-significantly gained weight.	[73]

Inland bearded dragons (n=8)	PO	20mg/kg SD	*Nannizziopsis guarroi*	Skin	-	NA	Plasma concentrations exceeded target MIC (0.03 µg/mL) by 4 hours after drug administration and remained above the target MIC for > 24 hours. Multiple-dosing studies on actual diseased bearded dragons are required to demonstrate efficacy.	No adverse effects were observed. 1 bearded dragon died but cause was attributed to iatrogenic trauma from gavage.	[74]
Northwestern pond turtle (n=18)	NEB	18mg (2mg/mL; 9mL) q24h x 28 days	*Emydomyces testavorans*	Shell	-	✓	All turtles had concentrations of terbinafine above MIC (> 0.06 µg/mL) in their keratin 1 week post final administration, as well as 2 week post final administration in the majority (75%) of turtles tested at this time point. Terbinafine also appears to resolve current lesions although it does not prevent recurrence in different shell locations.	No adverse effects were observed.	[75]
Northwestern pond turtle (n=7)	PO	30mg/kg SD	*Emydomyces testavorans*	Shell	-	NA	Average terbinafine plasma concentrations at steady-state were predicted using computational methods but actual studies were not conducted. Multiple-dosing studies on actual diseased turtles are required to demonstrate efficacy. Css(q24h): 469.2 ± 493.5 ng/mL Css(q48h): 234.4 ± 245.8 ng/mL	No adverse effects were observed.	[76]
Northwestern pond turtle (n=7)	BEC	30mg/kg SD	*Emydomyces testavorans*	Shell	-	NA	Average terbinafine plasma concentrations at steady-state were predicted using computational methods but actual studies were not conducted. Multiple-	No adverse effects were observed.	[76]

							dosing studies on actual diseased turtles are required to demonstrate efficacy. Css(q24h): 1,458.2 ± 1,939.9 ng/mL Css(q48h): 727.1 ± 967.5 ng/mL		
Red-eared slider turtle (n=6)	PO	15mg/kg SD	NR	NR	-	NA	Predicted steady-state 12-h concentration for terbinafine administered q12h was 0.07 μg/mL, which exceeds the terbinafine MICs (0.015–0.06 μg/mL) reported for Endomyces isolates. However, only 2 of the turtles were predicted to exceed the upper reported terbinafine MICs due to the wide intraspecies variabilities. Multiple-dosing studies on actual diseased turtles are required to demonstrate efficacy.	No adverse effects were observed.	[77]
Veiled chameleon (n =2)	PO	5 mg/kg q24h x 2 weeks	*Metarhizium viride*	Systemic	-	≈	Mean survival time of the treated chameleons was 32 months compared to the untreated chameleons (mean survival time = 1 month). Upon AST, all isolates (n=26) were resistant to 25μg of fluconazole and 8μg of itraconazole. 4 isolates were susceptible 20μg of amphotericin B and 1μg of voriconazole, with the rest of the isolates showing resistance. Most of the isolates (20/26) were susceptible to 100 IU of nystatin, 30 μg terbinafine, 50 μg clotrimazole and 5 μg posaconazole respectively.	NR	[78]

Veiled chameleon (n=1)	PO	5 mg/kg q24h x 7 weeks	*Metarhizium viride*	Systemic	-	≈	Mean survival time of the treated chameleons was 32 months compared to the untreated chameleons (mean survival time = 1 month).	NR	[78]
**POLYENES**									
**Amphotericin B**									
Greek tortoise (n=1)	Lung lavage via transcarapacial pulmonary catheter	0.1mg/kg q24h x 34 days	*Candida albicans*	Respiratory tract	-	✓	With treatment, dyspnea was minimal and an increase in weight with further decreases in AST, LDH and bile acids. By day 46, there was no evidence of respiratory disease.	No adverse effects were observed.	[79]
**Nystatin**									
Diamond python (n=1)	PO	1000,000 IU/kg q24h x 10 days	*Rhizopus sp.*	GI tract	-	✓	Fecal smears performed 2 and 4 weeks after treatment were negative for any fungal hyphae.	NR	[80]
Honduran milksnake (n=2)	PO	100,000 IU/kg q24h x 10 days	*Geotrichum sp., Trichosporon beigehi and Candida rugosa*	GI tract	-	✓	Stools were considered normal within 1 week after discontinuation of therapy. Repeat direct fecal smears performed 2 and 4 weeks after treatment did not show any fungal hyphae.	NR	[80]
Veiled chameleon (n=1)	PO	100,000 IU/kg q24h x 2 weeks	*Metarhizium granulomatis, Metarhizium viride*	Systemic	-	≈	Mean survival time of the treated chameleons was 32 months compared to the untreated chameleons (mean survival time = 1 month).	NR	[78]

Veiled chameleon (n=1)	PO	100,000 IU/kg q24h x 3 weeks	*Metarhizium granulomatis, Metarhizium viride*	Systemic	-	≈	Mean survival time of the treated chameleons was 32 months compared to the untreated chameleons (mean survival time = 1 month).	NR	[78]
**MACROLIDES**									
Natamycin									
Red-eared slider turtle (n=1)	PO	3mg/kg	*Candida tropicalis*	GI tract	-	≈	Wet preparations indicated a distinct decrease in yeast cells, and urine was less mucoid and clearer though general clinical status of the turtle remained more or less the same. Turtle died 20 days after the onset of symptoms. Shown to be in poor condition upon necropsy.	NR	[81]
Red-eared slider turtle (n=1)	PO	3mg/kg x 10 days	*Candida spp.*	GI tract	-	✓	At the end of 10 days, faeces returned to normal and were free of yeast cells.	NR	[81]

AST = aspartate aminotransferase; **BEC** = bioencapsulation into an earthworm vehicle; **CANV** = Chrysosporium anamorph of Nannizziopsis vriesii complex; **Cmax** = peak plasma drug concentration; **Css** = mean steady state plasma concentration; **IV** = intravenous; **LDH** = lactate dehydrogenase; **MIC** = minimum inhibitory concentration; **n** = number of animals; **NEB** = nebulization; **NA** = not applicable; **NR** = not reported; **PO** = oral; **ROA** = route of administration; **SC** = subcutaneous; **SD** = single dose; **SOI** = site of infection; ✓ = efficacy observed; ≈ = possible sign of efficacy but inconclusive; × = no efficacy observed.

[a]: Broad zones of inhibition were used as measure for antifungal susceptibility testing instead of MIC.

References

1. Joseph, V., D. Pappagianis, and D. Reavill, *Clotrimazole Nebulization for the Treatment of Respiratory Aspergillosis*. 1994.
2. Sanchez, C.R. and S.Z. Murray, *Diagnosis and successful treatment of a presumptive case of aspergillosis in a Micronesian kingfisher (Halcyon cinnamomina cinnamomina).* Avian Dis, 2005. **49**(2): p. 309-12.
3. Perelman, B., et al., *Use of azole compounds for the treatment of experimental aspergillosis in turkeys.* Avian Pathol, 1992. **21**(4): p. 591-9.
4. Flammer, K. and M. Papich, *Pharmacokinetics of fluconazole after oral administration of single and multiple doses in African grey parrots.* Am J Vet Res, 2006. **67**(3): p. 417-22.
5. Pericard, J.M., *Clinical assessment on the use of fluconazole per os in 24 African grey parrots (Psittacus erithacus): acceptance, side effects and efficiency*, in *Arles*. 2005, Proceedings of the 8th European Conference of the Association of Avian Veterinarians, 6th Scientific ECAMS Meeting of the European College of Avian Medicine and Surgery 222: France. p. 222-227.
6. Schunk, R.S.K., et al., *Multicentric Cryptococcosis in a Congo African Grey Parrot (Psittacus erithacus erithacus).* J Avian Med Surg, 2017. **31**(4): p. 373-381.
7. Maccolini É, O., et al., *A Disseminated Cryptococcus gattii VGIIa Infection in a Citron-Crested Cockatoo (Cacatua sulphurea citrinocristata) in Québec, Canada.* J Avian Med Surg, 2017. **31**(2): p. 142-151.
8. Ratzlaff, K., M.G. Papich, and K. Flammer, *Plasma concentrations of fluconazole after a single oral dose and administration in drinking water in cockatiels (Nymphicus hollandicus).* J Avian Med Surg, 2011. **25**(1): p. 23-31.
9. Raso, T.F., et al., *Cryptococcosis outbreak in psittacine birds in Brazil.* Med Mycol, 2004. **42**(4): p. 355-62.
10. Molter, C.M., J.R. Zuba, and R. Papendick, *Cryptococcus gattii osteomyelitis and compounded itraconazole treatment failure in a Pesquet's parrot (Psittrichas fulgidus).* J Zoo Wildl Med, 2014. **45**(1): p. 127-33.
11. Proença, L.M., et al., *Antemortem Diagnosis and Successful Treatment of Pulmonary Candidiasis in a Sun Conure (Aratinga solstitialis).* J Avian Med Surg, 2014. **28**(4): p. 316-21.
12. Smith, J.A., et al., *Effects of compounding on pharmacokinetics of itraconazole in black-footed penguins (Spheniscus demersus).* J Zoo Wildl Med, 2010. **41**(3): p. 487-95.
13. Zalesak, S.M., et al., *Preliminary Pilot Study of Itraconazole After a Single Oral Dose of a Veterinary Formulation Solution in African Penguins (Spheniscus demersus).* J Avian Med Surg, 2020. **34**(1): p. 52-56.
14. Orosz, S.E., et al., *Pharmacokinetic Properties of Itraconazole in Blue-Fronted Amazon Parrots (Amazona aestiva aestiva).* Journal of Avian Medicine and Surgery, 1996. **10**(3): p. 168-173.
15. Abrams, G.A., et al., *Aspergillus Blepharitis and Dermatitis in a Peregrine Falcon-Gyrfalcon Hybrid (Falco peregrinus x Falco rusticolus).* Journal of Avian Medicine and Surgery, 2001. **15**(2): p. 114-120.
16. Bonar, C.J. and A.H. Lewandowski, *Use of a Liposomal Formulation of Amphotericin B for Treating Wound Aspergillosis in a Goliath Heron (Ardea goliath).* Journal of Avian Medicine and Surgery, 2004. **18**(3): p. 162-166.
17. Bunting, E.M., et al., *Evaluation of oral itraconazole administration in captive Humboldt penguins (Spheniscus humboldti).* J Zoo Wildl Med, 2009. **40**(3): p. 508-18.
18. Gümüşsoy, K.S., et al., *Experimental Aspergillus fumigatus infection in quails and results of treatment with itraconazole.* J Vet Med B Infect Dis Vet Public Health, 2004. **51**(1): p. 34-8.
19. Rundfeldt, C., et al., *A model for treating avian aspergillosis: serum and lung tissue kinetics for Japanese quail (Coturnix japonica) following single and multiple aerosol exposures of a nanoparticulate itraconazole suspension.* Med Mycol, 2013. **51**(8): p. 800-10.
20. Wlaź, P., et al., *Activity and Safety of Inhaled Itraconazole Nanosuspension in a Model Pulmonary Aspergillus fumigatus Infection in Inoculated Young Quails.* Mycopathologia, 2015. **180**(1-2): p. 35-42.

21. Souza, M.J., P. Redig, and S.K. Cox, *Plasma Concentrations of Itraconazole, Voriconazole, and Terbinafine When Delivered by an Impregnated, Subcutaneous Implant in Japanese Quail (Coturnix japonica).* J Avian Med Surg, 2017. **31**(2): p. 117-122.
22. Sánchez, C.R., et al., *Population pharmacokinetics of itraconazole solution after a single oral administration in captive lesser flamingos (Phoeniconaias minor).* J Vet Pharmacol Ther, 2019. **42**(1): p. 1-6.
23. Rayment, K.M., et al., *Atypical Candidiasis of the Gnathotheca in a Lesser Flamingo (Phoeniconaias minor).* J Avian Med Surg, 2022. **35**(4): p. 457-463.
24. Tell, L.A., et al., *Studies on itraconazole delivery and pharmacokinetics in mallard ducks (Anas platyrhynchos).* J Vet Pharmacol Ther, 2005. **28**(3): p. 267-74.
25. Kano, R., et al., *Antifungal drugs-low-susceptibility in Aspergillus flavus isolated from a captively reared Okinawa rail (Hypotaenidia okinawae).* J Vet Med Sci, 2021. **83**(1): p. 28-30.
26. Jones, M.P., et al., *Pharmacokinetic Disposition of Itraconazole in Red-Tailed Hawks (Buteo jamaicensis).* Journal of Avian Medicine and Surgery, 2000. **14**(1): p. 15-22.
27. Anderson, N.L., *Candida/Megabacteria Proventriculitis in a Lesser Sulphur-Crested Cockatoo (Cacatua sulphurea sulphurea).* Journal of the Association of Avian Veterinarians, 1993. **7**(4): p. 197-201.
28. Flammer, K., et al., *Pharmacokinetics of voriconazole after oral administration of single and multiple doses in African grey parrots (Psittacus erithacus timneh).* Am J Vet Res, 2008. **69**(1): p. 114-21.
29. Hyatt, M.W., et al., *PHARMACOKINETICS OF ORALLY ADMINISTERED VORICONAZOLE IN AFRICAN PENGUINS (SPHENISCUS DEMERSUS) AFTER SINGLE AND MULTIPLE DOSES.* Journal of Zoo and Wildlife Medicine, 2017. **48**(2): p. 352-362, 11.
30. Hyatt, M.W., et al., *VORICONAZOLE TOXICITY IN MULTIPLE PENGUIN SPECIES.* J Zoo Wildl Med, 2015. **46**(4): p. 880-8.
31. Johnson, S.D., J.P. Buchweitz, and A.F. Lehner, *Single oral or intravenous administration of voriconazole achieved recommended therapeutic minimum inhibitory concentrations against Aspergillus in the common raven (Corvus corax).* Am J Vet Res, 2022. **83**(10).
32. Schmidt, V., et al., *Plasma concentrations of voriconazole in falcons.* Vet Rec, 2007. **161**(8): p. 265-8.
33. Azmanis, P., et al., *Pharmacokinetics of voriconazole after a single intramuscular injection in large falcons (Falco spp.).* Med Mycol, 2020. **58**(5): p. 661-666.
34. Tell, L.A., et al., *Efficacy of voriconazole in Japanese quail (Coturnix japonica) experimentally infected with Aspergillus fumigatus.* Med Mycol, 2010. **48**(2): p. 234-44.
35. Sadar, M.J., et al., *Mycotic Keratitis in a Khaki Campbell Duck (Anas platyrhynchos domesticus).* J Avian Med Surg, 2014. **28**(4): p. 322-9.
36. Parsley, R.A., et al., *Application of different pharmacokinetic models to describe and predict pharmacokinetics of voriconazole in magellanic penguins following oral administration.* J Vet Pharmacol Ther, 2019. **42**(1): p. 74-84.
37. Kline, Y., et al., *Pharmacokinetics of voriconazole in adult mallard ducks (Anas platyrhynchos).* Med Mycol, 2011. **49**(5): p. 500-12.
38. Beernaert, L.A., et al., *Designing a treatment protocol with voriconazole to eliminate Aspergillus fumigatus from experimentally inoculated pigeons.* Vet Microbiol, 2009. **139**(3-4): p. 393-7.
39. Carneiro de Castro, I.F., et al., *Safety of an Intravenous Formulation of Voriconazole as an Intramuscular Injection in Pigeons (Columba livia f. domestica).* J Avian Med Surg, 2022. **36**(3): p. 262-271.
40. Gentry, J., et al., *Voriconazole Disposition After Single and Multiple, Oral Doses in Healthy, Adult Red-tailed Hawks (Buteo jamaicensis).* J Avian Med Surg, 2014. **28**(3): p. 201-8.
41. Parsley, R.A., L.A. Tell, and R. Gehring, *Pharmacokinetics of a single dose of voriconazole administered orally with and without food to red-tailed hawks (Buteo jamaicensus).* Am J Vet Res, 2017. **78**(4): p. 433-439.

42. Rojo-Solís, C., et al., *Pharmacokinetics of single dose oral Terbinafine in common shelducks (Tadorna tadorna).* J Vet Pharmacol Ther, 2021. **44**(4): p. 510-515.
43. Evans, E.E., et al., *Pharmacokinetics of terbinafine after oral administration of a single dose to Hispaniolan Amazon parrots (Amazona ventralis).* Am J Vet Res, 2013. **74**(6): p. 835-8.
44. Nakeeb, S.M., B. Babus, and A.Y. Clifton, *Aspergillosis in the Peruvian Penguin (Spheniscus humboldti).* The Journal of Zoo Animal Medicine, 1981. **12**(2): p. 51-54.
45. Ward, F.P., D.G. Fairchild, and J.V. Vuicich, *Pulmonary aspergillosis in prairie falcon nest mates.* J Wildl Dis, 1970. **6**(1): p. 80-3.
46. Crispin, S.M. and K.C. Barnett, *Ocular candidiasis in ornamental ducks.* Avian Pathol, 1978. **7**(1): p. 49-59.
47. Redig, P.T. and G.E. Duke, *Comparative pharmacokinetics of antifungal drugs in domestic turkeys, red-tailed hawks, broad-winged hawks, and great-horned owls.* Avian Dis, 1985. **29**(3): p. 649-61.
48. Wolff, P.L., K.R. Petrini, and C. Kolmstetter, *An Outbreak of Aspergillosis in Crested Wood Partridges (Rollulus rouloul).* Journal of Zoo and Wildlife Medicine, 1992. **23**(1): p. 108-112.
49. Manharth, A., et al., *Disseminated Phaeohyphomycosis Due to an Exophiala species in a Galapagos Tortoise, Geochelone nigra.* Journal of Herpetological Medicine and Surgery, 2005. **15**(2): p. 20-26.
50. Innis, C.J., et al., *Single-dose pharmacokinetics of ceftazidime and fluconazole during concurrent clinical use in cold-stunned Kemp's ridley turtles (Lepidochelys kempii).* J Vet Pharmacol Ther, 2012. **35**(1): p. 82-9.
51. Mallo, K.M., et al., *Pharmacokinetics of fluconazole in loggerhead sea turtles (Caretta caretta) after single intravenous and subcutaneous injections, and multiple subcutaneous injections.* J Zoo Wildl Med, 2002. **33**(1): p. 29-35.
52. Eatwell, K., *Suspected fatal Chrysosporium anamorph of Nannizziopsis vriesii (CANV) dermatitis in an albino Boa constrictor (Constrictor constrictor).* J Small Anim Pract, 2010. **51**(5): p. 290.
53. Johnson, R.S., et al., *Deep fungal dermatitis caused by the Chrysosporium anamorph of Nannizziopsis vriesii in captive coastal bearded dragons (Pogona barbata).* Aust Vet J, 2011. **89**(12): p. 515-9.
54. Masters, N.J., et al., *Dermatomycosis caused by Paranannizziopsis australasiensis in five tuatara (Sphenodon punctatus) and a coastal bearded dragon (Pogona barbata) in a zoological collection in New Zealand.* N Z Vet J, 2016. **64**(5): p. 301-7.
55. Girling, S.J. and M.A. Fraser, *Treatment of Aspergillus species infection in reptiles with itraconazole at metabolically scaled doses.* Vet Rec, 2009. **165**(2): p. 52-4.
56. Lindemann, D.M., et al., *PHARMACOKINETICS, EFFICACY, AND SAFETY OF VORICONAZOLE AND ITRACONAZOLE IN HEALTHY COTTONMOUTHS (AGKISTRODON PISCIVORUS) AND MASSASAUGA RATTLESNAKES (SISTRURUS CATENATUS) WITH SNAKE FUNGAL DISEASE.* J Zoo Wildl Med, 2017. **48**(3): p. 757-766.
57. Lafortune, M., et al., *Shell and Systemic Hyalohyphomycosis in Fly River Turtles, Carettochelys insculpta, caused by Paecilomyces lilacinus.* Journal of Herpetological Medicine and Surgery, 2005. **15**(2): p. 15-19.
58. Van Waeyenberghe, L., et al., *Voriconazole, a safe alternative for treating infections caused by the Chrysosporium anamorph of Nannizziopsis vriesii in bearded dragons (Pogona vitticeps).* Med Mycol, 2010. **48**(6): p. 880-5.
59. Bowman, M.R., et al., *Deep fungal dermatitis in three inland bearded dragons (Pogona vitticeps) caused by the Chrysosporium anamorph of Nannizziopsis vriesii.* Med Mycol, 2007. **45**(4): p. 371-6.
60. Paré, J.A., et al., *Cutaneous mycoses in chameleons caused by the Chrysosporium anamorph of Nannizziopsis vriesii (Apinis) Currah.* J Zoo Wildl Med, 1997. **28**(4): p. 443-53.
61. Manire, C.A., et al., *Steady-state plasma concentrations of itraconazole after oral administration in Kemp's ridley sea turtles, Lepidochelys kempi.* J Zoo Wildl Med, 2003. **34**(2): p. 171-8.
62. Manire, C.A., et al., *Disseminated mycotic infection caused by Colletotrichum acutatum in a Kemp's ridley sea turtle (Lepidochelys kempi).* J Clin Microbiol, 2002. **40**(11): p. 4273-80.

63. Heatley, J.J., et al., *Fungal Periodontal Osteomyelitis in a Chameleon, Furcifer pardalis.* Journal of Herpetological Medicine and Surgery, 2001. **11**(4): p. 7-12.
64. Gamble, K.C., T.P. Alvarado, and C.L. Bennett, *Itraconazole plasma and tissue concentrations in the spiny lizard (Sceloporus sp.) following once-daily dosing.* J Zoo Wildl Med, 1997. **28**(1): p. 89-93.
65. Rajeev, S., et al., *Isolation and characterization of a new fungal species, Chrysosporium ophiodiicola, from a mycotic granuloma of a black rat snake (Elaphe obsoleta obsoleta).* J Clin Microbiol, 2009. **47**(4): p. 1264-8.
66. Page, C.D., et al., *Preliminary pharmacokinetics of ketoconazole in gopher tortoises (Gopherus polyphemus).* Journal of Veterinary Pharmacology and Therapeutics, 1988. **11**(4): p. 397-401.
67. Page, C.D., et al., *Multiple-Dose Pharmacokinetics of Ketoconazole Administered Orally to Gopher Tortoises (Gopherus polyphemus).* Journal of Zoo and Wildlife Medicine, 1991. **22**(2): p. 191-198.
68. Abarca, M.L., et al., *Cutaneous hyalohyphomycosis caused by a Chrysosporium species related to Nannizziopsis vriesii in two green iguanas (Iguana iguana).* Medical Mycology, 2008. **46**(4): p. 349-354.
69. Hellebuyck, T., et al., *Cutaneous hyalohyphomycosis in a girdled lizard (Cordylus giganteus) caused by the Chrysosporium anamorph of Nannizziopsis vriesii and successful treatment with voriconazole.* Vet Dermatol, 2010. **21**(4): p. 429-33.
70. Scheelings, T., et al., *Cutaneous and systemic mycoses from infection with Lecanicillium spp. in captive Guthega skinks (Liopholis guthega).* Australian Veterinary Journal, 2015. **93**(7): p. 248-251.
71. Wright, T.L., et al., *PLASMA VORICONAZOLE CONCENTRATIONS FOLLOWING SINGLE- AND MULTIPLE-DOSE SUBCUTANEOUS INJECTIONS IN WESTERN POND TURTLES (ACTINEMYS MARMORATA).* J Zoo Wildl Med, 2021. **52**(2): p. 538-547.
72. Innis, C.J., et al., *Plasma Concentrations and Safety Assessment of Voriconazole in Red-Eared Slider Turtles (Trachemys scripta elegans) after Single and Multiple Subcutaneous Injections.* Journal of Herpetological Medicine and Surgery, 2014. **24**(1-2): p. 28-35.
73. Kane, L.P., et al., *Pharmacokinetics of nebulized and subcutaneously implanted terbinafine in cottonmouths (Agkistrodon piscivorus).* J Vet Pharmacol Ther, 2017. **40**(5): p. 575-579.
74. McEntire, M.S., et al., *Single-dose pharmacokinetics of orally administered terbinafine in bearded dragons (Pogona vitticeps) and the antifungal susceptibility patterns of Nannizziopsis guarroi.* Am J Vet Res, 2021. **83**(3): p. 256-263.
75. Flaminio, K.P., et al., *Pharmacokinetics of Nebulized Terbinafine in Plasma and Keratin of Northwestern Pond Turtles (<i>Actinemys marmorata</i>) Associated with Emydomycosis.* Journal of Herpetological Medicine and Surgery, 2022. **32**(1): p. 48-55, 8.
76. Cerreta, A.J., et al., *Bioencapsulation is a feasible method of terbinafine administration in Emydomyces testavorans-infected western pond turtles (Actinemys marmorata).* Am J Vet Res, 2022. **84**(1).
77. Eshar, D., et al., *TERBINAFINE PHARMACOKINETICS FOLLOWING SINGLE-DOSE ORAL ADMINISTRATION IN RED-EARED SLIDER TURTLES (TRACHEMYS SCRIPTA ELEGANS): A PILOT STUDY.* J Zoo Wildl Med, 2021. **52**(2): p. 520-528.
78. Schmidt, V., et al., *Fungal dermatitis, glossitis and disseminated visceral mycosis caused by different Metarhizium granulomatis genotypes in veiled chameleons (Chamaeleo calyptratus) and first isolation in healthy lizards.* Vet Microbiol, 2017. **207**: p. 74-82.
79. Hernandez-Divers, S.J., *Pulmonary candidiasis caused by Candida albicans in a Greek tortoise (Testudo graeca) and treatment with intrapulmonary amphotericin B.* J Zoo Wildl Med, 2001. **32**(3): p. 352-9.
80. Raiti, P., *Use of Nystatin to Reduce Suspected Overgrowth of Enteric Fungal Organisms in a Diamond Python, Morelia spilota spilota and Two Honduran Milksnakes, Lampropeltis trianqulum hondurensis.* Bull Assoc Rept Amphib Vet, 1998. **8**: p. 4-8.
81. Zwart, P. and M. Buitelaar, *Candida tropicalis: Enteric infections and their treatment in chelonians.* 1980.
